# COVID-19 Vaccination Intention Associated with Behaviors towards Protection and Perceptions Regarding the Pandemic

**DOI:** 10.3390/jpm12020295

**Published:** 2022-02-17

**Authors:** Chrysoula Dafogianni, Polyxeni Mangoulia, Despoina Pappa, Panagiota Xanthopoulou, Ioannis Koutelekos, Mixalis Zografakis-Sfakianakis, Eftychia Ferentinou, Antigoni Fountouki, Marianna Drakopoulou, Anna Giga, Nikos Anastasiou, Nikoletta Margari, Georgia Fasoi

**Affiliations:** 1 Department of Nursing, University of West Attica, 12243 Athens, Greece; cdafog@uniwa.gr (C.D.); ikoutel@uniwa.gr (I.K.); mdrakopoulou@uniwa.gr (M.D.); agiga@uniwa.gr (A.G.); nmargari@uniwa.gr (N.M.); gfasoi@uniwa.gr (G.F.); 2 Department of Nursing Specialties and Education, Evangelismos General Hospital, 10676 Athens, Greece; pmangoulia@uniwa.gr; 3 Department of Nursing, University of West Attica & Henry Dunant Hospital Center, 12243 Athens, Greece; 4 Faculty of Social Sciences, Hellenic Open University, 10677 Patras, Greece; std124687@ac.eap.gr; 5 Department of Nursing, Hellenic Mediterranean University, 71410 Crete, Greece; mzografakis@hmu.gr; 6 Department of Nursing, University of West Attica & Children Hospital “Agia Sofia”, 12243 Athens, Greece; eferentinou@uniwa.gr; 7 Faculty of Nursing, International University of Greece, 57001 Thessaloniki, Greece; antifountou@yahoo.gr; 8 Central Clinic of Athens, 10680 Athens, Greece; nikos.anastasiou@gmail.com

**Keywords:** COVID-19 vaccination, protection, pandemic, attitudes

## Abstract

Background: The impressively rapid availability of different types of COVID-19 vaccines and, on the other hand, the degree of their effectiveness as opposed to the likelihood of serious or non-serious side effects place a fairly large percentage of the population at a crossroads regarding the choice to get vaccinated or not, hence threatening achievement of total immunization coverage and full immunity. This study aimed to assess COVID-19 vaccination intention in Greece regarding protection behaviors and perceptions of the pandemic. Methods: A total of 3753 participants completed a specially designed electronic questionnaire anonymously and voluntarily. The study population consisted of healthcare workers, students, members of professional societies, teachers, and professors. The questionnaire was composed of four parts pertaining to demographic data and possible changes in hygiene attitudes during the COVID-19 pandemic. Results: In total, 43.3% of the participants stated that SARS-CoV-2 poses a significant risk. The most widespread protection practice was avoiding crowded places (90.1%), followed by reducing the use of public transport (86.1%) and washing their hands with soap and water more often than usual (84.2%). Women undertook significantly more behavioral changes than men and participants of other nationalities. About half of the participants (44%) implemented seven behavioral changes. Lower personal and general perceived risk due to COVID-19 was significantly associated with lower intention to get vaccinated. Conclusion: Strong hesitancy was observed towards COVID-19 vaccination. There is a need for further studies to be conducted to investigate the benefits and safety of vaccines for the purpose of better informing the public. Healthcare personnel can and should play a key role in this process.

## 1. Introduction

In December 2019, an acute atypical respiratory disease emerged in Wuhan city, Hubei province in mainland China. It was soon discovered that a novel coronavirus (SARS-CoV-2) was responsible. The coronavirus outbreak was officially declared a pandemic by the World Health Organization (WHO) [1] in March 2020 (coronavirus disease 2019—COVID-19—WHO, 2021), posing a significant threat to global public health.

The COVID-19 pandemic, an unprecedented phenomenon in the modern world, has affected every aspect of human activity and life around the globe. It is caused by severe acute respiratory syndrome coronavirus-2, or SARS-CoV-2, with cases ranging from individuals who are asymptomatic to those who experience severe respiratory distress and pneumonia and may even die [2]. Furthermore, the lack of targeted therapy continues to be a serious problem. The number of deaths caused by the disease is rising constantly worldwide. Many countries have been forced to take extreme measures, such as social distancing and lockdowns [3]. On the other hand, public engagement in health-protective behaviors, including social distancing and hygiene-related behaviors, is considered crucial to reduce the risk of infection and successfully manage the outbreak. A very important impact of the pandemic was the mental status of general population. There was disorientation with regards to the constant public threat; the insecurity of people’s jobs; and the repetition of pandemic news through the TV, the Internet, and social media, especially during lockdown policy when communication was affected. Thus, emotions became complex and daily motivations became fewer [4,5,6].

In the absence of an effective drug against SARS-CoV-2, the efforts of the international scientific community and researchers have been focused on the development of novel, safe, and effective vaccines. Just nine months after COVID-19 was declared a pandemic, the first vaccine was officially approved by the European Medicines Agency (E.M.A) [7] and the U.S. Food and Drug Administration (FDA) in late 2020 (Pfizer-BioNTech COVID-19 Vaccine, 2021). Vaccination against COVID-19 is currently the only available long-term solution to coronavirus disease 2019. However, although vaccines that reduce symptomatic disease have a crucial role in reducing the burden of COVID-19, vaccine hesitancy is a major barrier to vaccine uptake and the achievement of community (herd) immunity, which is required to protect the population, especially the most vulnerable persons [8].

With vaccines against COVID-19 now available to the public and vaccination programs in progress internationally, understanding the development and determinants of COVID-19 vaccination intentions among the public is vital. Meanwhile, a well-organized vaccination program is currently considered the most effective strategy against the COVID-19 outbreak [9,10]. According to Faasse and Newby [11], data from previous emerging infectious disease outbreaks highlight the role of several key factors in shaping engagement in health-protective behaviors and vaccination intentions. Recent scientific research, while showing that vaccine hesitancy is a global problem, also points out that that there is a quite large amount of people willing to be vaccinated.

The present study aimed to investigate vaccination intention in Greece and assess the prevailing attitude towards protective behaviors. An investigation focusing on the perceptions of the Greek population regarding this pandemic is of extreme importance given the economic and social upheavals it has caused both in Greece and worldwide.

## 2. Materials and Methods

### 2.1. Participants

Of the 3753 participants who completed the questionnaire, 3 did not meet the inclusion criteria and 56 were rejected because they did not meet the quality check criteria. The final sample was thus composed of 3694 participants (response rate = 98.5%).

### 2.2. Data Collection

The present study is a cross-sectional study performed through completion of a questionnaire with closed-ended questions. Random sampling was conducted from December 2020 to January 2021 among healthcare personnel, students, teachers, professors, and members of professional associations. The highest number of cases was 2185 infections just in the beginning of the research. On 9 January 2021, the total deaths were 5227, where the last questionnaire of the study was completed. Due to the COVID-19 pandemic and national restrictive measures, distribution and completion of the questionnaire was carried out electronically through personal emails; the process was conducted anonymously and voluntarily, and appropriate approval was previously obtained from the participants. During the study, Greek citizens were already applied in a restrictive measure, the so-called “lockdown’’ for the second time. Schools were also closed and any sport activity was suspended. After final submission of the answers, the questionnaire was automatically included in the electronic data files of the study. Issues of research ethics were applied after study protocol acceptance by the competent committees of the participating universities. 

### 2.3. Instruments

In order to conduct the study, relevant questionnaires were employed to assess people’s perceptions of the pandemic and their intention regarding vaccination. Specifically, the questionnaire of Sherman et al. [12] was used to investigate attitudes and beliefs not only concerning other vaccines but also COVID-19 itself and COVID-19 vaccination, specifically. Furthermore, the questionnaire of Seale et al. [13], developed for vaccination against H1N1 in 2009, was also applied in this research project. The questions were assessed and adjusted appropriately. This tool consisted of four parts, as detailed below.

Firstly, demographic and personal data were recorded, namely, age, gender, education level, total family income, religion, and place of residence. 

The second part contained questions about participants’ beliefs concerning COVID-19 disease and, specifically, whether they considered it dangerous or not. The 10-point Likert scale was used to show the population’s level of agreement concerning specific tool items. 

The third part of the tool comprised 24 questions related to participants’ beliefs as to the possible advantages of COVID-19 vaccination, their reasons for mistrust of the vaccination, and their perceived self-efficacy and behavioral control. 

The final part included eight questions regarding behavior change due to the COVID-19 outbreak related to hygiene habits and daily activities. It would be useful to mention that during the research, the only available option for COVID-19 vaccination was the Pfizer/Biontech shot for healthcare professionals. At the final phase of the study, Greece obtained approval for the Moderna vaccine but it was deployed a few days later [14]. 

### 2.4. Data Analysis

Quantitative variables were expressed as the mean (standard deviation) or as the median (interquartile range). Qualitative variables were expressed as absolute and relative frequencies. Multiple linear regression analysis was conducted in order to find independent factors associated with the number of behavioral changes adopted due to the pandemic and participants’ intention to get vaccinated against COVID-19. 

Participants’ demographics and their perceptions of the COVID-19 pandemic were used as independent variables. 

In the event that the intention to get vaccinated was the dependent variable, implementing at least one behavioural change was entered in the analysis as an independent variable. Adjusted regression coefficients (β) (or beta coefficients) and their confidence intervals were derived from the analyses. Due to the large number of predictors in the model, statistical significance was set at *p* < 0.01. Assumptions about the analysis were checked. All reported p values were two-tailed. Analyses were conducted using SPSS statistical software (version 22.0).

## 3. Results

### 3.1. Demographic Characteristics

The sample consisted of 3697 participants (67.7% females), with a mean age of 29.4 years (SD = 12.6 years) from 2 December 2020 to 9 January 2021. Most of the participants were Greek nationals and Christian Orthodox, with the percentages being 96.5% and 76.8%, respectively (Table 1). In addition, 48.8% of the participants were high school graduates, 37.7% were key workers in the pandemic, and 24.1% had an annual income of up to EUR 10,000. Furthermore, 57.4% of the participants were living with two to three people in the same household and 70.3% were living in Athens. Lastly, 72.2% of the participants had not been vaccinated against seasonal flu during the previous winter.

### 3.2. Perceptions toward Protection and COVID-19 Vaccination Intention

As far as the perceived risk from COVID-19 for the population of Greece is concerned, 43.3% of the participants stated that it is significant (Table 2). Personal risk from COVID-19 was considered to be moderate among 27.7% of the participants. A total of 39.2% of the participants had not had, and did not currently have, COVID-19, and 70.6% personally knew someone who had had it or who currently had it. As mentioned above, total deaths from COVID-19 were 5227 already. Lastly, 36.4% of the participants thought that their employer would want them to have the COVID-19 vaccination, and 32.2% knew that there is currently a widely available vaccination to protect against the virus. However, the only choice of vaccine type in Greece during that period was Pfizer/Biontech for healthcare workers. Announcements from the Ministry of Health were about the imminent start of the vaccination rollout with the Moderna vaccine on February 2021 to the general population over 85 years old.

**Figure 1:**
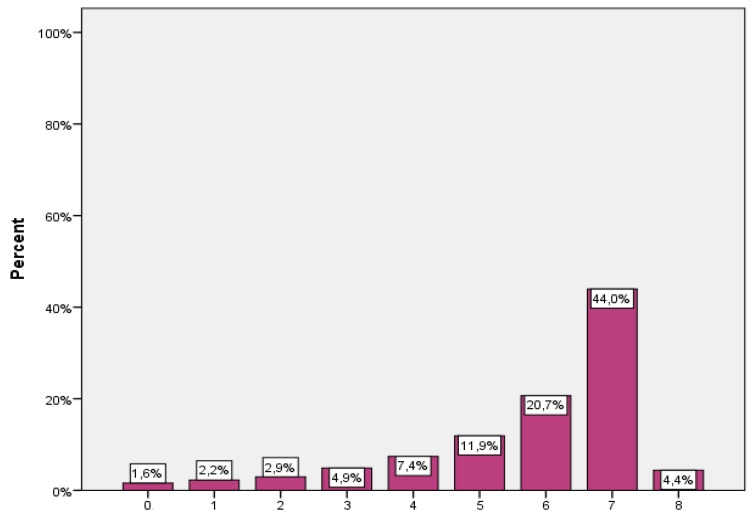
The number of behavioral changes due to the COVID-19 pandemic. The number of behavioral changes due to the COVID-19 pandemic.

Participants’ responses regarding their behavior due to the COVID-19 pandemic are presented in Table 3. The most common practice reported was avoiding crowded places (90.1%), followed by reducing the amount they use public transport and washing their hands with soap and water more often than usual (86.1% and 84.2%, respectively). Almost all the participants (98.4%) made at least one behavioral change due to the COVID-19 pandemic. The number of behavioral changes due to the COVID-19 pandemic ranged from 0 to 8, with the mean being 5.9 (SD = 1.7). About half of the participants (44%) implemented seven behavioral changes (Figure 1).

Women made significantly more behavioral changes compared to men (Table 4). On the other hand, participants who were not of Greek nationality and participants who were living abroad made significantly fewer behavioral changes. Moreover, age and the number of people living in the same household as the participants were significantly and positively associated with the number of behavioral changes adopted. Furthermore, as perceived general risk due to the pandemic decreased with the passage of time, the number of behavioral changes accordingly diminished significantly. As far as perceived personal risk was concerned, it was found that participants who thought they had minor or no personal risk undertook significantly fewer behavioral changes in comparison to those who felt that they ran a major personal risk. Knowing someone personally who previously or currently had COVID-19 was significantly associated with implementing more behavioral changes. 

The intention to get vaccinated against COVID-19 ranged from 0 (strongly disagree) to 10 (strongly agree), with the mean value being 5.6 (SD = 3.6) and the median being 6 (IQR: 2–9). A significantly lower intention to get vaccinated against COVID-19 was demonstrated by women and Christian Orthodox participants (Table 5). In contrast, participants living outside Athens had significantly greater intention to get vaccinated, as did those who had been vaccinated against seasonal flu during the previous year. Lower personal and general perceived risk due to COVID-19 was significantly associated with lower intention to get vaccinated. Those who personally knew someone who previously or currently had COVID-19 and those who made at least one behavioral change were significantly associated with higher intention to get vaccinated. 

## 4. Discussion

The present study investigated COVID-19 vaccination intention associated with behaviors towards protection and perceptions regarding the pandemic. Almost all of the participants undertook at least one behavioral change due to the COVID-19 pandemic and half of them seven behavioral changes, such as washing their hands more frequently with soap. The most common practice reported was avoiding crowded places, followed by reducing use of public transport and washing their hands with soap and water more often than usual. Female gender, age, and the number of people living in the same household as the participant were significantly and positively associated with the number of behavioral changes.

Similar results were presented by Dafogianni et al. [15] in a survey of 130 Greek nurses, whereby the great majority of participants reported that they apply measures to help prevent the spread of COVID-19 at home. This might be due to the fact that the majority of participants were married with children and faced a responsibility to protect their family members. In the same study, the most frequent coping strategies were acceptance of reality, serious consideration of the next step, and an attempt to see the situation differently and more positively or seeking something positive in the situation. Older age was more strongly correlated with active/positive coping.

The COVID-19 pandemic has globally imposed a heavy burden of disease on all countries and health systems, as there were no specific antiviral treatments for SARS-CoV-2 at the time [16,17,18]. As immunization is one of the most successful and cost-effective health interventions to prevent and control infectious diseases, vaccines against COVID-19 are of crucial importance [19,20]. Thus, countries worldwide are endeavoring to accelerate research into the development of COVID-19 vaccines. Around the world, there are now 135 COVID-19 vaccine candidates undergoing clinical trials and 194 candidates in pre-clinical development.

Previous reports on the acceptance and uptake of pandemic vaccines have shown unsatisfying results. For example, in the case of the 2009 H1N1 pandemic, the general public’s willingness to receive the H1N1 pandemic vaccine was reported to have ranged from 17% to 67% across studies from Australia, the U.S.A., Greece, the U.K., and France [21,22,23,24,25,26,27]. As concerns around vaccines used against new emerging pandemics, including the 2009 H1N1 pandemic, are novel, public concern about vaccine safety has frequently been identified as a serious barrier to vaccine acceptance [20,23,24,27,28,29], while attitudes and past history regarding vaccination, especially the history of influenza vaccination, were the main predictors of pandemic vaccine uptake [20,21,22,25,27,28,29,30,31,32].

In this study, the mean value of intention to be vaccinated against COVID-19 was 5.6±3.6. Male gender and living outside the city of Athens were positively correlated with the intention to get vaccinated against COVID-19. Perceived personal risk and knowing someone who had had or who currently had the virus were significantly associated with undertaking more behavioral changes and greater intention to get vaccinated. Women accounted for the vast majority of this study’s respondents, suggesting that COVD-19 vaccine hesitancy could be greater in real life settings.

In a study by Wang et al. [33] on 2058 participants in China, more than half of respondents wanted to get vaccinated as soon as possible, while others intended to delay the vaccination until they had confirmed the safety of the vaccine. Among respondents who accepted vaccination, the most significant factors influencing their vaccination acceptance were the following: male gender, marital status, risk perception, history of influenza vaccination, belief in COVID-19 vaccine efficacy, valuing one’s doctor’s recommendations, vaccination convenience, and vaccine price (where applicable, e.g., some early regional cases in China) [34,35]. On the other hand, the population in Greece then had the only choice for one type of vaccination, the mRNA vaccine. Several cases of adverse events followed by vaccination came up globally, worrying both scientists as well as people who were thinking of being vaccinated. Thrombosis, myocarditis, blood clots with low platelets, or even death were described in literature as possible side effects from vaccines. This situation increased population’s hesitancy surrounding mRNA vaccines, viral vectors vaccines, and other types of them that were finally approved after the study period [7]. 

In a multi-methods study in the U.K. on 1252 parents and guardians, most participants also reported they would like to receive COVID-19 vaccination for themselves and for their children. Participants who self-reported as Black, Asian, Chinese, mixed, or other ethnicity were almost three times more likely to reject a COVID-19 vaccine for themselves and their children than White British, White Irish, and other White participants [36]. This result is in contrast to that of the present study, in which Christian Orthodox participants had lower intention of getting vaccinated. However, the latter could be due to differences in the questions asked, highlighting the need for cross-country surveys and consistency in the wording of questions. Another reason could be the timeline in which the survey was conducted. Generally speaking, ethnicity and religious beliefs seem to play role in people’s intention to get vaccinated.

The present study also confirmed the positive role of influenza vaccination history and the belief in vaccine effectiveness in accepting immediate vaccination for COVID-19, which was consistent with that of previous studies [20,21,22,25,27,28,30]. Furthermore, respondents’ risk perception was an important predictor of vaccination acceptance, which was also found in other studies [31,34,35]. In the study by Detoc et al. [36] in 3259 individuals, male gender and older age were not associated with perceived individual risk, but rather with fears surrounding COVID-19. In the same vein, healthcare workers were more likely to get vaccinated or to participate in a vaccine clinical trial than the other participants. They hypothesized that healthcare workers are particularly vulnerable, while they were aware that, for instance, the latter social group accounted for 10% of those infected in Italy and in Greece [37,38]. Nurses and assistant nurses were less willing to accept vaccination against COVID-19 than physicians. Vaccine hesitancy was associated with a decrease in COVID-19 acceptance [39]. Although most medical students perceived the importance of the COVID-19 vaccine [39], almost half of the medical and nursing students were vaccine-hesitant [40,41,42]. 

In the present study, only one-third of the participants knew that there is currently a widely available vaccination to protect against SARS-CoV-2. Earnshaw et al.’s study [43], on 845 U.S. adults in April 2020, noted that one-third of participants believed one or more conspiracy theories about COVID-19. It was also found that intention to get vaccinated was 3.9 times lower among participants who believed in conspiracy theories, while these individuals additionally indicated less support for COVID-19 public health policies than participants who did not believe in conspiracy theories.

The aforementioned surveys show that the difference in vaccination acceptance varies greatly between countries, from around 62% in France to 80% in Denmark and the U.K. [44] and is reflective of trust in vaccines and healthcare systems more broadly as well as in governments [35]. Two recent U.S. studies on this issue indicated that the potential acceptance of the prospective COVID-19 vaccines ranged from 57.6% to 68.8% [45,46]. A recent global survey involving 19 countries reported a less than 55% acceptance rate in Russia, with the highest rate being in China at 90% [47]. Sallam et al. [48] reported that only 29.4% of the respondents in Jordan, Kuwait, and other Arab countries would get vaccinated against COVID-19. This rate, which appears to be among the lowest acceptance rates globally, is alarming [49].

Chevallier, Hacquin, and Mercier [50] offer three recommendations to increase COVID-19 vaccination rates, as follows: first, implementation of communication campaigns utilizing evidence-based levers and argumentation tools provided by experts; second, the use of behavioral insights to make vaccination more accessible; and third, helping early adopters to communicate about their decision to be vaccinated so as to accelerate the emergence of pro-vaccination norms.

Our study has several limitations. First, the online survey may have limited the representativeness of the study sample; however, in an attempt to address this problem, a large sample size and a random sampling method were used. Moreover, some self-reported answers may lead to information bias, while the study results may differ from real practice and may change over time. 

## 5. Conclusions

Vaccine hesitancy remains high during the current COVID-19 pandemic, with slow rates of vaccination being registered during the first half of 2021. Vaccine availability and efficiency towards the COVID-19 pandemic had a serious impact on vaccination intention. Several reasons have been stated by the participants for vaccine refusal during this period. It is evident that further studies are needed to investigate the levels of acceptance of COVID-19 vaccination and people’s perceptions during different phases of the pandemic and the reasons why vaccine efficacy and safety are not taken more seriously into consideration. Health professionals should be involved to support the public and help with informed decision making as regards acceptance of vaccinations. 

## Figures and Tables

**Table 1:** Sample characteristics.

		*n* (%)
Gender	Male	1193 (32.3)
	Female	2498 (67.7)
Age, mean (SD)		29.4 (12.6)
Nationality	Greek	3566 (96.5)
	Other	110 (3.0)
	Prefer not to say	18 (0.5)
Religion	Christian Orthodox	2821 (76.8)
	No religion	329 (9.0)
	Other	90 (2.4)
	Don’t know	1 (0.0)
	Prefer not to say	432 (11.8)
Educational level	Primary school	1 (0.0)
	High school	1802 (48.8)
	University	1155 (31.3)
	MSc degree	661 (17.9)
	Prefer not to say	70 (1.9)
Key worker		1395 (37.7)
Annual income	EUR ≤ 10,000	869 (24.1)
	EUR 10,001–20,000	783 (21.7)
	EUR 20,001–30,000	240 (6.6)
	EUR 30,001–40,000	104 (2.9)
	EUR 40,001–50,000	55 (1.5)
	EUR 50,001–75,000	35 (1)
	>75,000	28 (0.8)
	Don’t know	723 (20.0)
	Prefer not to say	776 (21.5)
Number of people in household	1	377 (10.2)
	2	684 (18.6)
	3–4	2114 (57.4)
	5–6	428 (11.6)
	>7	37 (1)
	Prefer not to say	43 (1.2)
Residence	Athens/Attica	2569 (70.3)
	Outside Athens	1036 (28.4)
	Abroad	48 (1.3)
Flu vaccination last winter	No	2665 (72.2)
	Yes	909 (24.6)
	Don’t know	104 (2.8)
	Prefer not to say	15 (0.4)

**Table 2:** Descriptive statistics for items measuring participants’ perceptions of the COVID-19 pandemic * not entered in the analysis.

Item	Level	*n* (%)
To what extent do you think COVID-19 poses a risk to people in Greece?	Major risk	1090 (29.5)
	Significant risk	1601 (43.3)
	Moderate risk	733 (19.8)
	Minor risk	220 (6.0)
	No risk at all	26 (0.7)
	Don’t know	25 (0.7)
To what extent do you think COVID-19 poses a risk to you personally?	Major risk	525 (14.2)
	Significant risk	884 (23.9)
	Moderate risk	1022 (27.7)
	Minor risk	997 (27.0)
	No risk at all	231 (6.3)
	Don’t know	37 (1.0)
Do you believe you have had, or currently have, COVID-19? *	Definitely	99 (2.7)
	Probably	269 (7.3)
	Probably not	1371 (37.1)
	Definitely not	1448 (39.2)
	Don’t know	504 (13.6)
	Prefer not to say	5 (0.1)
Do you personally know anyone (excluding yourself) who has had COVID-19?	No	1076 (29.2)
	Yes	2608 (70.6)
	Prefer not to say	9 (0.2)
As far as you know, would your employer want you to have the COVID-19 vaccination? *	No	184 (5.1)
	Yes	1315 (36.4)
	Don’t know	1634 (45.3)
	Prefer not to say	477 (13.2)
As far as you know, is there currently a widely available vaccination to protect against COVID-19? *	No	1550 (42.3)
	Yes	1180 (32.2)
	Don’t know	829 (22.6)
	Prefer not to say	107 (2.9)

**Table 3:** Behavioral responses to COVID-19 pandemic.

	*n* (%)
Washed my hands with soap and water more often than usual	3112 (84.2)
Used alcoholic hand gel more than usual	3013 (81.6)
Increased the amount I clean or disinfect things that I might touch, such as door knobs	2645 (71.6)
Kept away from crowded places generally	3343 (90.5)
Reduced the amount I use public transport	3180 (86.1)
Deliberately cancelled or postponed a social event, such as meeting friends, eating out, or going to a sports event	2893 (78.3)
Reduced the amount I go into shops	2980 (80.7)
Kept one or more of my children out of school or preschool	226 (42.6)
Undertook ≥ 1 behavioral change due to the COVID-19 pandemic	3637 (98.4)
Number of behavioral changes due to the COVID-19 pandemic, mean (SD)	5.9 (1.7)

**Table 4:** Multiple regression analysis results with number of behavioral changes due to the COVID-19 pandemic as dependent variable, and participants’ characteristics and their perceptions of the COVID-19 pandemic as independent variables. Note. The model was based on 2863 cases with complete data. * *p* < 0.01; ** *p* < 0.001.

	Unstandardized Coefficient β	Standardized Coefficient β	99% CI for β	*p* Value
Gender (reference: Men)	0.20	0.05	0.05 to 0.36	0.001 *
Age	0.01	0.07	0.00 to 0.02	0.001 *
Nationality (reference: Greek)	−0.79	−0.07	−1.23 to −0.34	<0.001 **
Key worker	−0.02	−0.01	−0.18 to 0.14	0.738
Religion (reference: No religion)				
Christian Orthodox	−0.09	−0.02	−0.32 to 0.15	0.343
Other	−0.01	0.00	−0.51 to 0.48	0.940
Number of people in household	0.09	0.05	0.01 to 0.18	0.005 *
Residence (reference: Athens/Attica)				
Outside Athens	0.07	0.02	−0.09 to 0.23	0.267
Abroad	−0.64	−0.04	−1.26 to −0.02	0.008 *
Flu vaccination last winter (reference: No)	0.10	0.03	−0.07 to 0.28	0.129
To what extent do you think COVID-19 poses a risk to people in Greece? (reference: Major risk)				
No risk at all/minor risk	−2.39	−0.34	−2.75 to −2.03	<0.001 **
Moderate risk	−1.09	−0.25	−1.35 to −0.83	<0.001 **
Significant risk	−0.33	−0.09	−0.52 to −0.13	<0.001 **
To what extent do you think COVID-19 poses a risk to you personally? (reference: Major risk)				
No risk at all/minor risk	−0.57	−0.15	−0.87 to −0.27	<0.001 **
Moderate risk	−0.09	−0.02	−0.36 to 0.18	0.382
Significant risk	0.06	0.02	−0.18 to 0.31	0.495
Do you personally know anyone (excluding yourself) who has had COVID-19? (reference: No)	0.24	0.06	0.08 to 0.40	<0.001 **

**Table 5:** Multiple regression analysis results with intention to get vaccinated against COVID-19 as a dependent variable, and participants’ characteristics, their perceptions of the COVID-19 pandemic, and adopting at least one behavioral change due to the COVID-19 pandemic as independent variables. Note. The model was based on 2863 cases with complete data. * *p* < 0.01; ** *p* < 0.001.

	Unstandardized Coefficient β	Standardized Coefficient β	99% CI for β	*p* Value

Gender (reference: Men)	−0.55	−0.07	−0.88 to −0.23	<0.001 **
Age	−0.01	−0.02	−0.02 to 0.01	0.294
Nationality (reference: Greek)	0.71	0.03	−0.22 to 1.63	0.050
Key worker	−0.06	−0.01	−0.39 to 0.26	0.616
Religion (reference: No religion				
Christian Orthodox	−1.35	−0.13	−1.83 to −0.86	<0.001 **
Other	−0.58	−0.03	−1.6 to 0.44	0.141
Number of people in household	−0.01	0.00	−0.19 to 0.17	0.914
Residence (reference: Athens / Attica)				
Outside Athens	0.36	0.05	0.03 to 0.69	0.005 *
Abroad	0.28	0.01	−1.01 to 1.57	0.576
Flu vaccination last winter (reference: No)	1.17	0.14	0.81 to 1.53	<0.001 **
To what extent do you think COVID-19 poses a risk to people in Greece? (reference: Major risk)				
No risk at all/minor risk	−4.96	−0.34	−5.72 to −4.2	<0.001 **
Moderate risk	−3.14	−0.34	−3.67 to −2.6	<0.001 **
Significant risk	−1.48	−0.20	−1.88 to −1.07	<0.001 **
To what extent do you think COVID-19 poses a risk to you personally? (reference: Major risk)				
No risk at all/minor risk	−1.04	−0.13	−1.66 to −0.42	<0.001 **
Moderate risk	−0.80	−0.10	−1.36 to −0.24	<0.001 **
Significant risk	−0.44	−0.05	−0.95 to 0.07	0.025
Do you personally know anyone (excluding yourself) who has had COVID-19? (reference: No)	0.44	0.05	0.1 to 0.78	0.001 *
Have undertaken at least one behavioral change due to COVID-19	1.39	0.05	0.11 to 2.68	0.005 *

## Data Availability

All the data generated during this study are included in this published article.
